# Revisiting the 1986 Molecular Cloning of Interleukin 6

**DOI:** 10.3389/fimmu.2014.00456

**Published:** 2014-09-23

**Authors:** Toshio Hirano

**Affiliations:** ^1^Osaka University, Suita, Osaka, Japan

**Keywords:** interleukin 6, molecular cloning of IL-6, history of IL-6 discovery, inflammation, autoimmune diseases, rheumatoid arthritis

We reported molecular cloning of B cell stimulatory factor-2 (BSF-2)/interleukin 6 (IL-6) in 1986 (1), the same year that IL-4 and IL-5 were cloned (2, 3). Prior to the publication of our article (1), it was known that antigenic stimulation induced growth and differentiation of B lymphocytes into antibody forming plasma cells with the help of T lymphocytes and this function of T lymphocytes could be replaced by soluble factors. Dutton in 1971 and Schimpl and Wecker in 1972 reported the presence of soluble factors that induced immunoglobulin production in B lymphocytes in the absence of T lymphocytes (4, 5). Schimpl and Wecker named the putative factor “T cell replacing factor” (TRF). In addition, Kishimoto and Ishizaka reported a soluble factor that enhanced IgE antibody production (6), while Takatsu and his colleagues reported a factor that enhanced anti-hapten antibody production (7). The molecular characteristics of these soluble factors, however, were unknown. Furthermore, other reports showed the possibility that there might be several kinds of soluble factors that affected the biological activities of B lymphocytes differently (8, 9). For example, Paul and his colleagues showed that the culture supernatant of mouse thymoma EL4 in combination with submitogenic doses of anti-IgM antibodies stimulated the proliferation of B lymphocytes without inducing antibody production (10). The putative factor was called “BCGF-I” or “BSF-1.” Swain and Dutton showed that culture supernatants from a long-term alloreactive T cell line, DL, induced growth in dextran sulfate stimulated B lymphocytes and BCL1 B cell tumors. The same culture supernatant when combined with IL-2 induced another response: antibody production in B cells (11). This putative factor was called “BCGF-II.” Thus, prior to our article, little was known about the molecular nature of the factors responsible for B lymphocyte stimulation, which is why many immunologists reported each factor by a different name based on its biological activity.

After studying at A. Nordin’s lab in the United States National Institutes of Health from 1973 to 1976, I returned to Y. Yamamura’s lab at Osaka University Medical School, where we showed that PWM-stimulated human T lymphocytes produced soluble factor(s) that induced immunoglobulin production in human B lymphocytes (12). In 1978, I moved to Osaka Prefectural Habikino Hospital where I saw many patients with tuberculous pleurisy. What I found there was that purified protein derivative (PPD)-stimulated pleural effusion cells from these patients produced soluble factor(s) that induced immunoglobulin production in PWM-stimulated human B lymphocytes (13). This finding led me to start purifying the putative factor in 1978. In 1981, Kishimoto’s group showed the presence of a soluble factor that enhanced IgG production in an Epstein–Barr virus transformed human B cell line, CESS. This putative factor was named “TRF” (14). We showed that the culture supernatant of PPD-stimulated pleural effusion cells obtained from patients with pulmonary tuberculosis or PWM-stimulated tonsillar mononuclear cells induced IgG production in a variety of Epstein–Barr virus-transformed B lymphoblastoid cell lines (15), which we called a “TRF-like factor” or “B cell differentiation factor II” (BCDFII) (15–17). We showed that the TRF-like factor/BCDFII was recovered in the fractions corresponding to the molecular weights 22 and 36 kDa by gel filtration, and that it had an isoelectric point between 5 and 6 (15).

In early 1984, I joined Kishimoto’s lab as an Associate Professor at Osaka University and continued purifying the putative factor from the culture supernatant of the HTLV-transformed human T cell line, TCL-NA1. We soon thereafter determined the sequence of its 14 N-terminal amino acids with the help of S. Tsunasawa, Institute for Protein Research, Osaka University at the end of 1984. The purified material had molecular weights of 19 and 21 kDa as identified by NaDodSO4/PAGE under reduced as well as non-reduced conditions. The IgG-inducing activity was found in the fractions corresponding to the molecular weights noted above and the isoelectric point ranged from 5.0 to 5.1 (18). We concluded that this material, which we called “BCDF” or “BSFp-2,” had properties what were similar to those of the TRF-like factor/BCDFII (15). Importantly, the purified protein did not exhibit any of the activities of IL-1 or -2, BCGF-I/BSF-1, BCGF-II or interferon (18).

Then we started the molecular cloning of the purified protein, using a probe based on information about the N-terminal partial amino-acid sequence with the kind help of T. Taniguchi, Osaka University. However, this procedure was much harder than I expected. Because of the many false positive clones bound to the probe, we were unable to obtain the cDNA encoding of the molecule with the identified N-terminal amino-acid sequence in 1985. This caused us great consternation, and we worried whether the identified sequence was correct. During this period, I suffered severe arrhythmia, which I attribute to the stress of the project. At the very beginning of 1986, I decided to purify the protein using newly obtained culture supernatants from TCL-NA1 cells. This time, instead of obtaining the N-terminal partial amino-acid sequence of the purified protein, I decided to acquire several fragments of the purified protein by digesting the protein with lysylendopeptidase, following the thoughtful advice of S. Tsunasawa. There was a serious risk of losing all the purified protein, however, due to the additional process of separating the fragmented peptides with high performance liquid chromatography. Nonetheless, our previous failures convinced me, we had little choice. On February 20 of that year, Honjo and his colleagues reported the molecular cloning of murine IgG1 induction factor, or IL-4 (2). However, I remained firm in the need to isolate the cDNA encoding of our purified protein. We were lucky to have obtained several protein fragments during the additional purification step, which eventually led us to successfully obtain their partial amino-acid sequences by the end of March 1986. We selected three highly reliable N-terminal partial amino-acid sequences from among eight fragments to make synthetic oligonucleotide probes, which we then used to clone the cDNA. On Sunday morning of May 25, 1986, I finally found one cDNA clone out of about 30,000 that miraculously – at least in my opinion – was bound by all three probes. Thus, after 8 years of hard work, beginning in 1978 when I started the purification of helper T cell factors at Osaka Prefectural Habikino Hospital, I had finally obtained the cDNA encoding of the protein inducing immunoglobulin in B cells, which we called “BSF-2” at that time. From the cDNA sequence, we predicted that the mature form of BSF-2 is composed of 184 amino acids and has an isoelectric point of 5, again, very similar to the TRF-like factor/BCDFII (15). This result was published in a November issue of *Nature* in 1986 (1) together with a report on the molecular cloning of IL-5 (3). The sequence of BSF-2 was found to be identical to that of an IL-1-induced 26-kDa protein with unknown biological activity, which was reported in the September issue of the *European Journal of Biochemistry* (19) and the sequence of IFN-β2, which was reported in the October issue of the *EMBO Journal* (20). In addition, the plasmacytoma/hybridoma/myeloma growth factor and the hepatocyte-stimulating factor were found to be identical to BSF-2 (21–25). At a nomenclature meeting in New York chaired by W. E. Paul at the end of 1988, the community agreed to call this molecule “IL-6” (26).

Our project originally began with the desire to isolate a factor responsible for stimulating B lymphocytes to produce immunoglobulin. What we found was something far more complex. IL-6 is a multifunctional cytokine that plays roles in immune responses, inflammation, hematopoiesis, and the endocrine and nervous systems, even though it has no interferon activity (27–29). Additionally, we revealed that IL-6 might be involved in autoimmune diseases and inflammatory diseases (30–33), opening the way for new therapies that regulate inflammatory diseases (34–37).

## Conflict of Interest Statement

The author declares that the research was conducted in the absence of any commercial or financial relationships that could be construed as a potential conflict of interest.
